# ‘All hands-on deck’, working together to develop UK standards for public involvement in research

**DOI:** 10.1186/s40900-020-00229-y

**Published:** 2020-09-16

**Authors:** Sally Crowe, Ade Adebajo, Hothan Esmael, Simon Denegri, Angela Martin, Bob McAlister, Barbara Moore, Martin Quinn, Una Rennard, Julie Simpson, Paula Wray, Philippa Yeeles

**Affiliations:** 1 Oxford, UK; 2 NIHR INVOLVE, Sheffield University & Barnsley Hospital NHS Trust, Barnsley, UK; 3 NIHR Central Commissioning Facility, Twickenham, UK; 4 NIHR National Director for Patients, Carers and the Public, London, UK; 5 Research and Development Division, Welsh Government, Cardiff, UK; 6 Health and Care Research Wales Public Involvement Delivery Board, Cardiff, UK; 7 Health and Care Research Wales Support and Delivery Centre, Cardiff, UK; 8 Public Health Agency, Belfast, Northern Ireland; 9 NIHR INVOLVE, Southampton, UK; 10 Chief Scientist Office, Edinburgh, Scotland

**Keywords:** Patient and public involvement, Research, Standards, Partnership, Collaboration, Improvement, Framework

## Abstract

**Background:**

Public involvement in research is an established part of the research process in the UK, however there remain questions about what good public involvement in research looks and feels like. Until now public involvement practitioners, researchers and members of the public have looked for answers in examples shared across networks, published case studies, guidance and research articles. Pulling these strands together, the UK Standards for Public Involvement provides six statements (standards) about public involvement in research. They were produced by a partnership of organisations from Scotland, Northern Ireland, Wales and England with contributions from involvement practitioners, public partners, researchers and research funders.

**Main body:**

Each standard has reflective questions, which are designed to encourage standard users to use approaches and behaviours that improve involvement, over time. The standards are designed to be used as a practical tool, and reflect the agreed hallmarks of good public involvement in research for example, flexibility in approaches used, shared learning, and mutual respect.

The standards development process is described from the initial idea and scoping, via the appraisal of existing standard sets and integration of values and principles in public involvement in research. The collaborative writing process of and consultation on the draft standard set is described, together with what changed as a result of feedback. The initiation of a year-long testing programme with forty participating research organisations, the experiential feedback and the resulting changes to the standards is summarised.

**Conclusion:**

This commentary paper describes, in some detail, a process to develop a set of six standards for public involvement in research in the UK. Producing a complex, national public involvement initiative is not without its challenges, and in supplementary material partnership members reflect on and share their experiences of standards development. The next phase of integration and implementation is explored with concluding comments from those that tested and helped improve the standards.

## Plain English summary

Public involvement in research, whereby members of the public get involved in different aspects of research, is part of research processes in the United Kingdom (UK). Examples of public involvement could be in research design, recruitment to a study or sharing research findings widely. However there remain questions about what good public involvement in research looks and feels like, especially to members of the public that get involved. Until now, people have looked for answers in examples shared across research and involvement networks and published guidance and research articles. The UK Standards for Public Involvement aim to provide that picture of good public involvement. Six statements (standards) with reflective questions for each, encourage standard users to adopt approaches and behaviours that improve public involvement in research, over time. They reflect the agreed hallmarks of good public involvement in research for example, flexibility in approaches used, shared learning, and mutual respect.

The standards were produced by a partnership of organisations from Scotland, Northern Ireland, Wales and England with contributions from public involvement practitioners, public partners, researchers and research funders. Working together on a complicated national public involvement in research initiative has been challenging. The development process is described from start to finish with additional material containing partnership members reflections on being involved. The next phase of widespread use and implementing the standards is considered, with contributions from those that tested the draft standards.

## Background

This commentary article is a description of how UK Standards for Public Involvement in research were developed by a partnership comprising professional and public contributors from Health and Care Research Wales, the National Institute for Health Research (England), Public Health Agency (Northern Ireland) and the Chief Scientist Office (Scotland). It describes the context for, and development process of the standards from 2015 to 2019.

Public involvement is a key part of UK research with increasing numbers of the public getting involved at different stages of the research process [1]. The partnership worked with the NIHR INVOLVE definition of public involvement in research, “research being carried out ‘with’ or ‘by’ members of the public rather than ‘to’, ‘about’ or ‘for’ them” [2]. There are other words and phrases that indicate and describe different approaches in public involvement in research e.g. patient and public engagement, participation, and co-production. The use of ‘public involvement’ in the standards, and in this article acknowledges these different perspectives and practice.

Organisations in the Standards Development Partnership fund research and have multiple reasons for supporting and enabling public involvement in research. It is helpful to understand these drivers as they shape how the partnership worked together and influenced the content of the standards. It can be a point of principle that public funded research requires public scrutiny and oversight, for example The National Institute for Health Research (NIHR) state that the public has a *“right to have a say in what and how publicly funded research is undertaken*” [3]. Similarly, Health and Care Research Wales describe their “*ambition is to create an environment where the public are central to health and social care research in Wales”* [4].

Partner organisations also see value in public involvement that improves research relevance, the Chief Scientist Office in Scotland *“places great importance on ensuring that the work that it funds is relevant for patients”* [5]. There is broad agreement in the partnership that public involvement can lead to improvements in research design and delivery [6] for example in research recruitment [7].

The context for public involvement in research changes over time, both in the structures that support it and what we collectively understand about it. The aspirations, hopes and ‘vision’ for public involvement in research also changes over time [8, 9]. Ongoing critiques of conflicting ideology, politics and tokenism in public involvement [10, 11] suggest a complex backdrop in which to develop standards. The publication of the NIHR strategy ‘Going the Extra Mile’ [12], which makes recommendations for public involvement in NIHR suggested standards for public involvement in research *“so that organisations across the NIHR see their adoption as integral to their continuous improvement in public involvement”* Whilst this gave NIHR license to take a leadership role in standards development, there was also a principle for the process to *“be co-produced with the public and other partners”.*

Standards are ‘processes, actions, or procedures that are deemed essential by authority, custom, or general consent’ Dickerson and Mayo Wilson suggest [13]. This definition stresses the need for standards to operate at organisational, and political levels as well as be culturally embedded and work for those that they are designed for. The authors warn that whilst standards can be useful in science and in life, adopting them can be time consuming and potentially expensive and there may be a ‘strong incentive to maintain the status quo rather than adopt new standards’. These conclusions suggest that any exercise to develop standards needs to bring potential standard users into the process to challenge cultural and political norms and ensure the end product is useful and works from a user perspective. The decision to test the draft standards for a year acknowledges the time needed to make organisational and cultural changes.

Standards in health care services and research are common, and have been in circulation for many years [14]. A review of 13 evaluations of standards used in mostly acute care (hospital) settings for health care improvement suggested there was a lack of agreement and evidence about the best ways to develop standards, for example choosing which words to use [15]. The partnership reviewed and drew from learnt experience of existing standard sets developed for public involvement in healthcare research/healthcare in the UK. As they paved the way for the partnership, we called them ‘path-finders’, they provided a rich and detailed resource. A summary is provided in Table 1, more information is available on the standards website [22].

**Table 1:** Pathfinder Standard Sets

Standards	Context	Key features	Comments
Draft standards for good practice in public involvement in research 2014 [16]	Developed as part of wider project. Public Involvement Impact Assessment Framework (PiiAF)	29 Standards; covering three stages; Beginning, Maintaining and Ending involvement	Built on principles for public involvement. Developed by Universities of Lancaster, Liverpool and Exeter.
National Involvement Standards (4PI), 2013 [17]	For use in mental health and wellbeing services	Five domains; principles, purpose, presence, process and impact, each with a range of sub headings.	Co-produced by National Service User Network. Reviewed 2015/16 (Reality and Impact Project)
The Scottish National Standards for Community Engagement 2005 [18]	For use in participation and community engagement in Scotland	7 Standards; Inclusion, Support, Planning, Working together, Methods, Communication and Impact	Reviewed in 2015 to reflect policy and legislation changes in community empowerment in Scotland. Build on experience.
The Northern Ireland Health and Social Care Personal and Public Involvement Standards 2015 [19]	Designed for use across social and health care.	5 Standards; Leadership, Governance, Opportunities (for support and involvement), Knowledge and skills, Measuring outcomes	Built on core values and principles (2007) Developed with Patient and Public Involvement Forum
Values and Standards for Patient Involvement in Health Technology Assessment [20, 21]	For specific use in Clinical Guidance and Health Technology Assessment (HTA) development	5 values; Relevance, Fairness, Equity, Legitimacy, Capacity Building. 5 quality standards for HTA processes generally and 5 for individual HTA’s	Developed by The Health Care Technology Assessment International Coalition Adopted by National Institute for Health and Clinical Excellence (NICE)

It is interesting to note the differences between the standard sets in size (numbers of standards) and depth (levels of detail for each standard covered). There were consistent overlapping themes in focus, use of one-word names for standards and deploying reflective questions. A review of one of the path-finders questioned whether standards are a “vehicle for conversation”, or a “template for perfection” [23]? The partnership preferred the former of these two approaches, wanting to strike a balance of a description of minimum expectations and critical reflection.

Notions of compliance or adherence to standards was also explored. Examples reviewed tended to be ‘aspirational’ by providing compelling arguments for use, rather than being explicit about what would happen if they weren’t met. The partnership agreed that ultimately it wasn’t for standard developers to dictate how people and organisations should use the standards once they were finalised. We did agree that the final standards should indicate what good quality public involvement in research might look and feel like, from the perspectives of involved public, researchers and others. In line with some of pathfinder examples they should encourage reflection and discussion on core areas of public involvement. Perhaps the most useful indicators of how useful standards are in improving public involvement in research will be evaluations and case studies, for example where the National 4PI Standards have been used in quality improvement [24] and strategic public involvement [25].

### Foundations for standards development

**Fig. 1:**
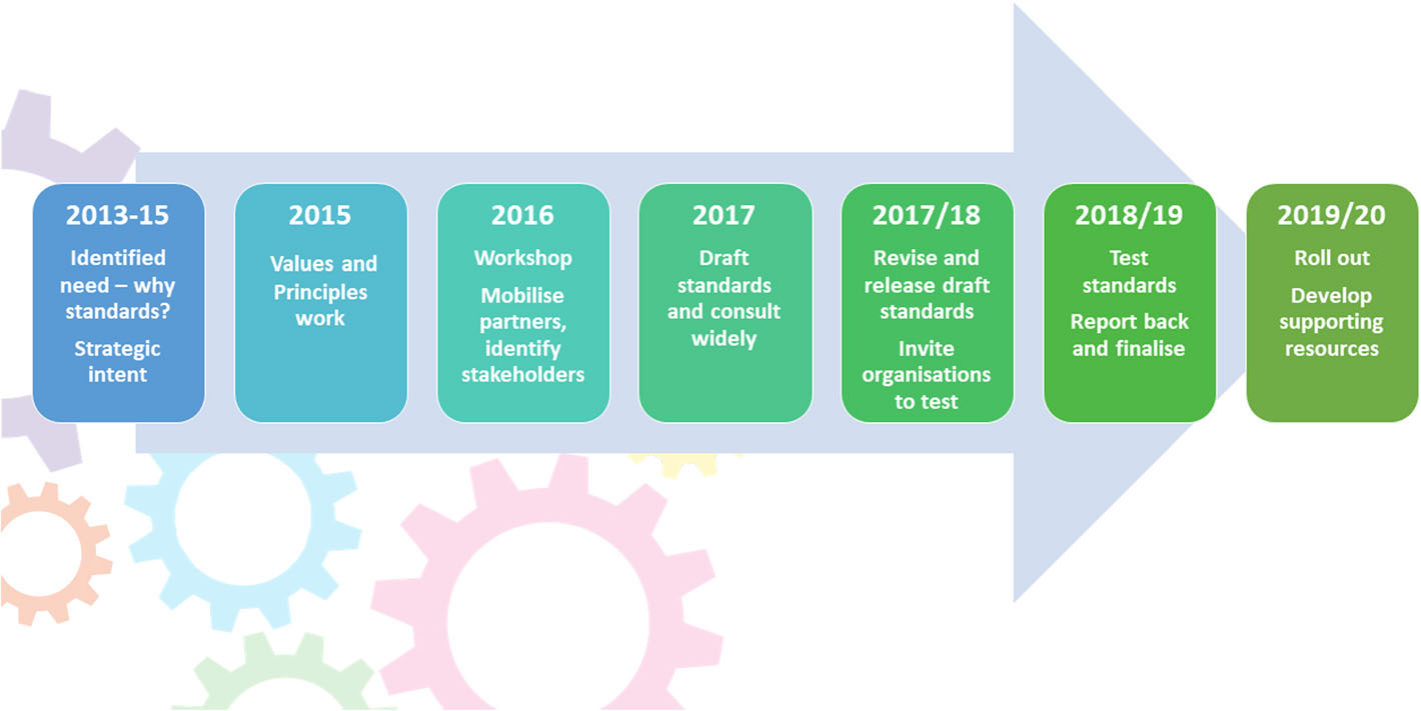
Timeline for UK Standards for Public Involvement

The development process of the standards is summarised below and this is reflected in the article (Fig. 1).

Partners identified their motivations and commitment to developing UK standards. They provided funds, in-kind contributions and agreed a development plan. Project support was provided by independent consultants.

An exploratory workshop in March 2016 brought together 47 participants including people with responsibility for leading and supporting public involvement in research, members of the public involved in research, people who had been involved in standards development (public involvement and other) and observers. Participants reflected on; public involvement values and principles, the meaning and purpose of standards; developmental and application issues, and critiqued different types of standards [26]. Participants were enthusiastic for standards that could support a shared understanding of good practice in public involvement in research. However, fears associated with encouraging a ‘tick box’ public involvement culture and loss of research funding if standards are not met were expressed by participants. It is important to acknowledge these reservations, and these were reflected in a critical article published midway through the process [27].

Workshop participant feedback provided important pointers for standards development;

- Keep them simple and easy to use in practice, for everyone
- Address minimum expectations of good public involvement
- Include a ‘stretch’ element for users that are more experienced in public involvement
- Encourage reflection and continuous improvement
- Distinguish between standards for activity and those for impact
- Ensure they appeal to a range of research organisations

Workshop findings provided a pragmatic touch point for standards development, and many workshop participants continued their involvement

### Standards development process

#### Step 1. Reviewing

Two sets of values and principles (NIHR INVOLVE and Health and Care Research Wales) [28] were combined to provide a framework in which to assess the pathfinder examples, Table 2.

**Table 2:** Public Involvement Values and Principles and UK Standards for Public Involvement

INVOLVE Values and Principles	Health Care Research Wales Principles	UK Standards
**Respect** Researchers, research organisations and the public respect one another’s roles and perspectives	Advocating **respect,** so researchers and the public show mutual respect for each other’s roles and perspectives and all parties are recognised and acknowledged for their contributions	**Working Together**
**Support** Researchers, research organisations and the public have access to practical and organisational support to involve and be involved	**Supporting** public involvement and engagement, ensuring researchers and the public access to the support necessary to enable them to involve and be involved;	**Support and Learning**
**Transparency** Researchers, research organisations and the public are clear and open about the aims and scope of involvement in the research	Promoting **transparency** in relationships between the public and researchers as well as transparency in Health and Care Research Wales Public Delivery Board decision making;	**Working Together** **Communications** **Governance**
**Responsiveness** Researchers and research organisations actively respond to the input of public members involved in research		**Communications**
**Fairness of opportunity** Researchers and research organisations ensure that public involvement in research is open to individuals and communities without discrimination	Encouraging **diversity** so involvement and engagement occurs with relevant groups with **equal opportunity**, and inclusivity	**Inclusive Opportunities**
**Accountability** Researchers, research organisations and the public are accountable are accountable for their involvement in research and to people affected by the research	Demonstrating **accountability**, of Health and Care Research Wales but also researchers to the communities and members of the public they involve and engage in their work	**Governance**
	**High quality and meaningful** public involvement in both Health and Care Research Wales, and in health and social care research more widely	**Impact**
	Encouraging appropriate public involvement and engagement **throughout the research process**, from setting research priorities through to dissemination of research	**Inclusive Opportunities**

Existing standards used in healthcare, community work and research were identified by searching the literature and following up leads and suggestions from colleagues. A sub set of examples were considered for an appraisal, Table 1.

Partnership members appraised three examples each, results of these were combined into a synthesis enabling the similarities and differences across the standard sets to be seen easily.

As none of the examples met all the criteria for a general UK set of standards it was decided to combine their most useful and generalisable aspects especially as there were clear overlaps in the topics. A whole day meeting of all partners, including public members, enabled in depth discussion, ‘post it’ notes were used to group key considerations. Six standards were agreed and key areas to be explored and acknowledged within each standard identified (italics);

- **Inclusive opportunities** – *inclusion and diversity, range of opportunities, research cycle*
- **Working together** – *aspects of co-production, clarity of expectations, roles and responsibilities, respect*
- **Support and learning** – *mechanisms for developing knowledge and skills, increasing effectiveness of involvement*
- **Communications** – *plain language, reporting, feedback, transparency, dissemination*
- **Impact** *– making a difference, developing systems for collecting information about impact*
- **Governance** – *accountability, leadership, management*

The synthesis results informed the framework for writing the standards, including; a one-word title, a one sentence explanation (what) and a rationale for each standard (why). Indicators and examples provided detail and context. Existing opportunities (e.g. NIHR Public Involvement Leads Meeting [29], and the Wales Involving People Network Annual Meeting) enabled very early ideas and preliminary text to be discussed and feedback was provided.

#### Step 2. Writing and communicating 

In early 2017 the partnership agreed the final wording for the standards and smaller sub-groups drafted supporting information for each one. There was a great deal of discussion about how directive and descriptive this should be, with consideration given to using reflective questions, referencing research cycles and ‘timelines’ e.g. research question generation, commissioning, planning and delivery. When writing the standards, the group acknowledged that they are interrelated and interdependent, mirroring the complexity of research, and public involvement in research.

Alongside standard writing, partnership members engaged with a wide range of people interested in the development process. We called these people stakeholders. A ‘Standards Network’ was created (400 plus members) and this became the primary way of providing updates and presenting opportunities for people to take part. The network was broad, and included; public (expert by experience, deeply involved patient, lived experience, lay rep, survivor, Patient Research Ambassador), academics (researcher, PhD student, clinical academic, service user researcher), involvement practitioners and managers (facilitator, PPI lead, Public Involvement Manager, Head of Public Engagement), research managers, administrators and project staff in public and charitable organisations. Additionally, NHS, local government and public oversight organisations such as Healthwatch were represented.

Partners from NIHR Central Commissioning Facility created a website in March 2017 to host information, introduce the project team and act as a transparent repository for documents. Social media enabled sharing progress, developments and encouraging feedback. A visual brand for the standards was agreed, with NIHR INVOLVE providing design input. Healthcare Research Wales ensured that the standards were available in Welsh, to comply with government requirements.

#### Step 3. Consulting

The purpose of the consultation was to facilitate an external appraisal of the draft standards and encourage ideas for improvement. A stakeholder map and plan was agreed, with each partner taking responsibility for priming their public and research networks and contacts. An online consultation survey with a mix of scoring and free text responses was piloted and refined to enable individual and group submissions.

The consultation package consisted of the draft UK Standards for Public Involvement (with early design ideas incorporated), an explanatory slide set and the consultation questions. The consultation launched on 30th June 2017 with a closing date of 1st September 2017. At the midpoint survey responses were reviewed, helping to identify which stakeholder groups needed encouragement to respond. The need for an ‘Easy Read’ version was highlighted in a Twitter exchange. Easy Read is text presented in an accessible format e.g. using short sentences, and images to represent key points, where possible. This omission was addressed and the deadline extended to October 2017 to allow for responses to the Easy Read version. A ‘tweet chat’ in the last week of the consultation window helped raise awareness and provide another route for feedback. The survey closed with 677 online, and three Easy Read responses.

The quantitative consultation results were compiled and appraised by partnership members and a Public Summary Report produced [30]. The public (patients, service user, carer) comprised 57.3% of individual responses, with academics (researcher, service user researcher), charity, healthcare and public involvement practitioners comprising 42.7%. For the group responses 23% were from research organisations, 23% from patient, service user and carer groups,15% from academic institutions, and the rest representing charities and ‘other’.

The qualitative results were independently assembled and analysed by Research Design Service North East, and a report produced [31]. There was consensus in feedback specifically; more use of plain language and simpler sentences; words such as ‘meaningful’, ‘competence’, and ‘confidence’ were considered open to interpretation, and phrases such as ‘community of interest’ and ‘visibility of power sharing’ confused people and needed changing. Sometimes it proved hard to settle on one word or phrase that responded to the feedback *and* was still in plain English.

There was little challenge to the content of the six standards or indicators, but there were suggestions for more flow to the standards, and a more coherent story. Examples in the indicators needed to reflect a wider variety of contexts for public involvement in research. There was valuable feedback on the ‘introduction to the standards’ section and suggestions to develop resources to support standards implementation.

#### Step 4. Rewriting

Health and Care Research Wales hosted two rewrite meetings with Partnership members in January 2018. In small writing groups consultation feedback was considered with each group presenting their suggested changes for a whole group sign off. Changes included; one new indicator added and one removed (due to overlap), rewriting examples to reflect a broader research canvas e.g. biomedical research, and creating a better flow with re-ordered indicators. A more detailed background and context section was developed and resources to support implementation added.

Examples of changes in the standards are shown in the first two columns of Table 3. These were displayed at the NIHR INVOLVE conference in November 2017, for a sense check and feedback.

**Table 3:** Changes to wording of UK Standards for Public Involvement over time

Standard	Before consultation	After consultation	After testing
**Inclusive Opportunities**	We provide clear, meaningful and accessible opportunities for involvement, for a wide range of people across all research.	We offer public involvement opportunities that are accessible and that reach people and groups according to research needs	Offer public involvement opportunities that are accessible and that reach people and groups according to research needs
**Working Together**	We create and sustain respectful relationships, policies, practices and environments for effective working in research.	We work together in a way that values all contributions, builds and sustains mutually respectful and productive relationships.	Working together in a way that values all contributions, and that builds and sustains mutually respectful and productive relationships
**Support and Learning**	We ensure public involvement is undertaken with confidence and competence by everyone.	We offer and promote support and learning which builds confidence and skills for public involvement in research.	Offer and promote support and learning opportunities that build confidence and skills for public involvement in research
**Communications**	We provide clear and regular communications as part of all involvement plans and activities	We use plain language for timely, two way and targeted communications, as part of involvement plans and activities.	Use plain language for well-timed and relevant communications, as part of involvement plans and activities
**Impact**	We assess report and act on the impact of involving the public in research.	To drive improvement, we capture and share the difference that public involvement makes to research and to the people involved.	Seek improvement by identifying and sharing the difference that public involvement makes to research.
**Governance**	We ensure the community of interest voices are heard, valued, and included in decision making. We implement, report and are accountable for our decisions.	We involve the public in our governance and leadership so that our decisions promote and protect the public interest.	Involve the public in research management, regulation, leadership and decision making.

#### Step 5. Launching

The *draft* standards were launched in March 2018, with communications teams from the four nations co-ordinating materials, a social media strategy, and a press release. A short video [32] and a blog [33] were shared on social media. Two workshops at a ‘Patients First’ conference hosted by the Association of Medical Research Charities and the Association of the Pharmaceutical Industry, and a presentation at the Involving People Network Annual Meeting 2018 in Cardiff completed launch activities.

#### Step 6. Testing

Concurrent to the launch preparation, researchers, groups and organisations were asked to submit an expression of interest to test the draft standards over one year [34]. It was important for a broad range of groups and research organisations to do this task. Applicants indicated which standards they wanted to implement and their organisational capacity and resources to support the testing. From 47 applications, ten ‘test beds’ (Additional file 1) were selected using the criteria in Table 4.

**Table 4:** Selection criteria for ‘test bed’ organisations

A geographical spread across the UK	
Different types of organisations - e.g. voluntary sector, medical charities, public sector, user-led, industry	
Different types of research interests - e.g. early phase research, implementation research, public health, clinical, mental health, technology	
Different knowledge and experience of public involvement in research, from novice to highly proficient	
Different availability of resources for public involvement from minimal to well resourced.	

Due to the level of interest in testing the standards unsuccessful applicants were offered the opportunity to be part of the testing community but without any formal support. Thirty accepted and they became known as ‘freestylers'. Freestylers and Test Beds agreed to take part in a ‘before and after’ testing survey. The partnership was not assessing testers on their public involvement, but finding out how well, or not, the standards helped them do and improve public involvement in research.

The organisations and contexts for testing varied, from large public sector programmes supporting public involvement in numerous healthcare studies, networks and collaborations of research and patient organisations, to single organisations and projects. Similarly, some testers were experienced in public involvement in research and others were in earlier stages of development. Some testers conducted public involvement mainly remotely or online, one planned to use the standards as an evaluation framework (of public involvement in research) and another as part of PhD studies.

A workshop in April 2018 brought together the ten testing teams (of professional and public members) with partners to explore plans, ideas and reservations. The partnership resisted the temptation to tell them how to go about implementing the draft standards, and experimentation was encouraged. Test bed sites were matched on a topic and/or geographical basis, encouraging future link ups. The Scottish and Welsh test-beds and freestylers met informally with the support of the Chief Scientist Office in Scotland and Health and Care Research Wales.

A series of teleconferences hosted by Health and Care Research Wales enabled progress, frustrations and learning to be shared, monitoring information about progress was shared ahead of teleconferences. A Google + community provided an online platform for resource sharing, but not everyone used this.

#### Step 7 Finalising

'After testing' perspectives were gathered in Spring of 2019, all 40 testers completed the survey. The evaluation [35] concluded that testers generally used the draft standards as a framework for public involvement supporting reflective practice and plans for future activities; as an audit /mapping tool to identify gaps and areas for improvement; and for support and reassurance that they were working towards achieving best practice.

Whilst the feedback was positive, testers suggested improvements to the standards including; clarity about ‘ownership’ of the standards; stressing the importance of context for standard use; indicating the importance of organisational support for standards implementation; a clearer description of standards purpose; reviewing the use of indicators *and* examples and improvements to language.

Test bed teams and the partnership gathered at a final workshop in London in May 2019 to share experiences and celebrate the completion of testing. There were presentations about achievements, challenges and what teams had to change most to implement the standards. A live illustrator captured the discussion themes.

Combining the survey and workshop feedback resulted in final changes to the standards and these included; contextual ‘framing’ e.g. emphasis on *improvement over time* rather than *meeting all the standards all the time*; repurposing the indicators into reflective questions; removal of the examples which were considered restricting; and further simplification of language.

The final standards were released in November 2019 [36], at INVOLVEfest in Northern Ireland. A recommendation from testers to supply real life examples from implementing the standards has been developed and is available as a booklet of Implementation Stories [37].

## Conclusions

Initial workshop feedback and appraisal of existing standards sets in public involvement suggested there was room for UK Standards for Public Involvement in research. A limitation in our approach was not commissioning a literature review to inform development prior to starting the process. The project was commissioned as part of strategic initiatives in English and Welsh public involvement in research, with its own momentum. Consideration of the advantages and disadvantages of different methods to devise the standards was less well developed than it could have been.

By working with previous standard developers, we benefitted from the learnt experience model, rather than what has been published. It would have been easier to write more than six standards, but there was a clear directive from the initial consultation workshop to keep it simple and focussed. Despite this, the collaborative writing process resulted in six standards that were probably trying to do the job of twelve, requiring major redrafts and simplification following the wider consultation and testing.

The value and strength of working as a UK-wide partnership cannot be overemphasised. People from four research organisations collaborating across national, organisational and cultural boundaries, working with their stakeholders to co-develop and test a set of standards proved challenging at times. However, the partnership is greater than the sum of its parts. It has thrived on mutual respect, healthy challenge, embracing difference, collective accountability, trust, good humour and endeavouring to model the standards in its own actions and behaviours. The results of an exercise to assess how the partnership had met these expectations is described in Additional Information file 2.

The future of the standards depends on two issues; widespread adoption and use and a broader, refreshed UK partnership as ‘custodian’ of the standards, and overseeing developments to them. Standard testers had things to say about this future phase, both as groups with an active interest in developing and improving their public involvement and as ‘early adopters’ of the standards. They do not represent the whole community of public involvement in research, but are likely to be influential in how the standards are interpreted more widely. They suggest that UK Standards for Public Involvement is a long-term commitment, identifying what works best in implementation will require time and on-going investment. Recognising that public involvement takes place in a wide variety of settings, and valuing the differences in how the standards may work in these, will be crucial to their success and ongoing development. At the time of writing, there is commitment from the current iteration of the partnership to monitor the standards and continue to encourage feedback from standard users.

## Supplementary information

**Additional file 1.** Organisations selected to test the draft Standards for Public Involvement.

**Additional file 2.** Reflections from partnership members.

## Data Availability

The datasets generated and/or analysed during the current study are available in the UK Standards for Public Involvement repository, https://sites.google.com/nihr.ac.uk/pi-standards/home

## Abbreviations

NIHR: National Institute for Health Research
UK: United Kingdom
