# Supplementary material for: ‘All hands-on deck’, working together to develop UK standards for public involvement in research
Source: Res Involv Engagem. 2020 Sep 16;6:53. doi: 10.1186/s40900-020-00229-y (PMC7493420; doi:10.1186/s40900-020-00229-y)
Supplement: Supplementary file 1 — Additional file 1. Organisations selected to test the draft Standards for Public Involvement. [file 40900_2020_229_MOESM1_ESM.docx]

**Additional Information File 1.**

**Organisations that tested the draft UK Standards for Public Involvement between 2018 and 2019.**

- Asthma UK Centre for Applied Research Patient and Public Involvement Platform, University of Edinburgh
- Research Institute for Primary Care and Health Sciences, Keele University
- Research Priorities Project, Royal College of Speech and Language Therapists
- The Centre for Ageing and Dementia Research (CADR) and the Wales School for Social Care Research
- Northern Ireland Cerebral Palsy Register, Queen’s University, Belfast
- Kidney Patient Involvement Network (KPIN)
- School of Medicine, Dentistry and Nursing (Nursing and Health Care), University of Glasgow
- Clinical Research & Innovation Office, Sheffield Teaching Hospitals NHS Foundation Trust
- Public Programmes Team, Manchester University NHS Trust, NIHR Manchester Biomedical Research Centre, NIHR Manchester Clinical Research Facilities
- Patient & Public Involvement Team, Royal College of Obstetricians & Gynaecologists
