# Supplementary material for: ‘All hands-on deck’, working together to develop UK standards for public involvement in research
Source: Res Involv Engagem. 2020 Sep 16;6:53. doi: 10.1186/s40900-020-00229-y (PMC7493420; doi:10.1186/s40900-020-00229-y)
Supplement: Supplementary file 2 — Additional file 2. Reflections from partnership members. [file 40900_2020_229_MOESM2_ESM.docx]

**Additional File 2. Reflections from partnership members**

**Inclusive Opportunities**

From the outset, the partnership thought about and planned to involve a wider community of people active and interested in public involvement in research. Internally we sought to be flexible and responsive in our working arrangements, especially for those living with or caring for people with long term conditions. This included holding partnership meetings in different locations, smaller topic-based group meetings, teleconferences and using an online platform to develop shared documents. Externally we made available many different opportunities for public involvement in the initiation and consultation phases including; an exploratory workshop, online and face to face consultation events, a drop-in session and workshops at related conferences, an independent public panel to assess the consultation feedback and social media interaction via a ‘tweet chat’. We addressed barriers to involvement. For example, at the initial workshop public members were offered payment for their time, travel costs (or upfront purchase of tickets) and other needs were catered for, such as overnight stays. Sometimes the pressure of deadlines meant that we didn’t always achieve our ambition to be inclusive. For example, the draft standards consultation ran over nine weeks in Summer 2017. This clearly inconvenienced some responders, and a handful of people told us that they couldn’t respond within the timeframe.

**Working Together**

Our purpose for involvement was clear, we functioned well as a group, reached decisions by consensus, and surfaced difficult topics. Membership stayed consistent and people attended and contributed throughout the process. Whilst there weren’t roles descriptions for each member of the partnership, there was a shared understanding of each members strengths and expertise. One member commented *“as a public member of the partnership I feel that everyone has an equal voice, and does not think about the rank and status of everyone in the room.”* The authors reflected on our experiences working together and identified the following attributes that enabled us to flourish; playing to the strengths and skills of different members, deploying good humour and getting to know each other as people first, embracing critical reflection especially from our public members who often kept us grounded and focussed, having trust and confidence in each other to reach the goal and independent and expert facilitation of meetings and teleconferences.

**Support and Learning**

There were realistic financial resources allocated to support public involvement in the development process, and the budget was reviewed regularly. Our learning was informal and ‘on the project’ rather than more structured learning opportunities. For example, in our Northern Ireland Public Health Agency partner we accessed insights and guidance from their experience of developing and implementing Personal and Public Involvement Standards for commissioning, service development and delivery of services. This helped us make decisions about consultation requirements, the need for a structured and extensive testing period, and the promotion and adoption of standards in real life. One of our public members had experience of developing standards in the charitable sector and this proved invaluable in discussions and decisions. Both public members of the partnership were paired with a known colleague from their partner organisation, allowing ongoing support and communication to happen external to the partnership ‘business’. This paper is evidence of our commitment to building on our learning and sharing it with others.

However, the frenetic pace of standards development sometimes prevented the partnership in capitalising on, or reflecting on learning from each stage of development. For example, in the pre consultation phase design work, that a sub group took forward (designing a questionnaire for multiple audiences, requiring rigor, structure, accessibility and plain language) was ‘lost’ in the rush to meet deadlines.

**Communications**

From the start, establishing and maintaining good internal and external communications was critical to the success of developing the standards *with* the active involvement of the organisations, groups and individuals who would eventually be using them. Having a ‘stakeholder map’ and a communications plan proved essential when working across so many organisations. Ongoing interaction with the wider research community was vital, to share progress, canvas opinions and encourage suggestions for improvement to the standards. This included communicating with the Standards Network, sharing a short update on progress after each partnership meeting, providing an up to date slide deck for use, and using hashtags on social media to locate and respond to conversations about the standards. We picked up a critical tweet about our omission to produce an Easy Read version of the draft standards, we were grateful for this and were able to correct our error.

The initial writing style of the first draft of the standards was too complex and in ‘management speak’, after review and redraft there was more positive feedback.

**Impact**

This standard is an interesting one for the partnership to reflect on, as the impact of the standards in public involvement in research cannot be reported on yet. However, we can consider the impact of public involvement on the process thus far. We can demonstrate that considerable changes were made to the standards pre and post consultation. As well as making the standards clearer and less managerial in tone, more contextual information was provided and resources developed for the testing phase. There have been signs of impact of the draft standards in requests from a variety of UK and international organisations wanting to adopt and use them. We noted public involvement job adverts referencing the standards and have evidence of research funders using the standards as a template to address quality improvement in public involvement in research. As part of the consultation we asked *‘what difference, if any, will it make to you to have a set of national standards for public involvement in research’*. The analysis of responses to this question categorised impacts in positive and productive ways (process, legitimacy of public involvement), but also potential negative, questionable and ‘conditional’ impacts (resource dependent, implementation challenges). This gives a valuable starting point for future evaluation of impact.

**Governance**

The partnership comprised four national government funded research organisations with professional and public membership, and a considerable investment of resources. Members of the partnership had responsibility within their own organisations to regularly report on progress and next steps. Duplication of effort was avoided and accurate documentation of decisions, developments and financial information allowed for scrutiny and critique from those inside and outside the partnership. The two public members of the standards development group were central to the partnership and process. Similarly, with 57% of responses to the draft standard consultation from the public there was equal value and weight given to public perceptions of the standards. Transparency was achieved by making regular progress reports, and consultation results publicly available.
